# Zoster sine herpete complicated by central nervous system infection in an immunocompetent adult: A case report

**DOI:** 10.5339/qmj.2025.118

**Published:** 2025-12-14

**Authors:** Khaled Zammar, Aisha Habib Ahmed, Liaquat Ali, Abeer Safan

**Affiliations:** 1 Neurology Department, Neuroscience Institute, Hamad Medical Corporation, Doha, Qatar; 2 Islamic International Medical College, Riphah International University, Rawalpindi, Pakistan; 3 Weil Cornell Medicine, Education City, Ar-Rayyan, Qatar *Email: kzammar@hamad.qa

**Keywords:** Zoster sine herpete, herpes zoster virus, varicella-zoster, meningitis, shingles, headache

## Abstract

**Background:**

Varicella zoster virus (VZV) reactivation can occur without the characteristic rash, a condition known as zoster sine herpete (ZSH). This case report highlights a rare presentation of VZV reactivation.

**Case presentation:** We present the case of a 40-year-old immunocompetent woman with a five-day history of headache, followed by neuropathic dermatomal pain localized to the left C2–C3 dermatomes. Cerebrospinal fluid analysis demonstrated a positive polymerase chain reaction (PCR) for VZV, confirming a diagnosis of ZSH complicated by VZV meningitis.

**Discussion:** The patient’s condition necessitated intravenous acyclovir therapy for 21 days. VZV meningitis can lead to severe outcomes, highlighting the importance of prompt diagnosis and timely treatment to prevent complications. The absence of a rash in ZSH necessitates heightened clinical suspicion to avoid potentially life-threatening consequences of VZV reactivation.

**Conclusion:**

Early recognition and prompt intervention are crucial for managing ZSH and preventing severe complications such as VZV meningitis, highlighting the importance of clinical awareness of atypical presentations of VZV reactivation among healthcare providers.

## 1. INTRODUCTION

Shingles, also known as herpes zoster (HZ), results from the reactivation of the varicella zoster virus (VZV), which remains dormant in the dorsal root ganglia after a primary varicella (chickenpox) infection. It presents as a vesicular eruption on an erythematous base, accompanied by severe dermatomal neuralgic pain. However, in less common cases, the VZV reactivation may occur as dermatomal neuralgia without a rash, a condition known as zoster sine herpete (ZSH).^1^ HZ and ZSH share the same pathophysiology and occur more frequently in immunocompromised patients. In the absence of a rash, ZSH is more likely to be misdiagnosed or missed, and patients may experience prolonged herpetic or post-herpetic neuralgia.^1,2^ The main reason is the continuous activation of VZV in the dorsal root ganglia, resulting in further damage to the affected nerves. This is mainly attributed to the delayed initiation or absence of antiviral treatment.^1,2^

In relatively rare cases, VZV reactivation may present as a central nervous system (CNS) infection, such as meningitis or encephalitis. In cases of HZ, the usual clinical presentation primarily includes fever, headache, nuchal rigidity, and altered sensorium. Interestingly, the absence of a rash in ZSH cases makes diagnosis more challenging.^2^

The case described in this report was managed in Qatar, where the healthcare setting is located in a region with high VZV vaccination coverage.

Routine varicella vaccination has significantly reduced the incidence of primary VZV infections, thereby decreasing the overall burden of HZ and its associated complications in vaccinated populations. However, vaccination rates vary widely across different regions and age groups. In countries with well-established immunization programs—such as the United States, Canada, Australia, and parts of Europe—childhood varicella vaccination is included in national immunization schedules, leading to a substantial decline in primary varicella infections and, subsequently, a lower incidence of HZ among younger populations.^3^ Conversely, in regions where varicella vaccination is not part of routine immunization, including some low- and middle-income countries, VZV infections remain more prevalent, thereby increasing the risk of HZ and its associated complications later in life.^4^

Although vaccination coverage is high in some areas, breakthrough HZ cases continue to occur, especially among older adults and immunocompromised individuals. Additionally, differences in vaccine uptake, healthcare access, and public health policies may affect the overall burden of VZV-related diseases. Cultural and societal factors also influence HZ outcomes, such as healthcare-seeking behaviors, symptom awareness, and access to antiviral therapy vary across populations.^2,3^

We report a previously healthy adult who developed VZV meningitis following ZSH involving the C2–C3 dermatomes. This case underscores the importance of maintaining a high index of suspicion for atypical VZV presentations, even in highly vaccinated populations, to ensure timely diagnosis and management. This case report was conducted with the required institutional approval from the Medical Research Center at Hamad Medical Corporation (reference number: MRC-04-24-477), and written informed consent was obtained from the patient to use their data and clinical images for publication.

## 2. CASE PRESENTATION

A 40-year-old female patient with no prior medical history presented to the Emergency Department (ED) with a new-onset headache of five days’ duration. The headache (HA) was holocranial and throbbing in nature, radiating to the neck, and significantly impairing her daily activities, rated 8/10 on the pain scale. It was associated with bilateral eye pain, blurry vision, left ear tinnitus, nausea, and vomiting. The HA was exacerbated by lying flat in bed, and paracetamol provided minimal relief of symptoms. There was no phonophobia, photophobia, sensory disturbances, weakness, diplopia, loss of consciousness, or abnormal movements. She was in her usual state of health prior to presentation, with no recent trauma or changes in lifestyle, sleep pattern, or weight. She was not using birth control medication, and her last menstrual period occurred 10 days prior. Additionally, she had no recent infections, exposure to sick contacts, or contact with toxins. There was no prior history of chronic headaches, and family history was notable for migraine headaches in her maternal and paternal aunts (second-degree relatives). The patient was a lifelong non-smoker and denied alcohol consumption or illicit drug use. She also denied recent use of any medications or herbal supplements and had no history of recent travel.

On physical examination, vital signs were within the normal range: temperature 36.8°C, pulse rate 82/min, respiratory rate 20/min, blood pressure 120/78 mmHg, and oxygen saturation 100% on room air. Neurologic examination showed normal mental status and intact cranial nerves, with no rashes or hyperalgesia. Fundoscopic examination was unremarkable. Motor, sensory, cerebellar, and gait examinations were all normal.

Her basic laboratory results in the ED, including a complete blood count and basic metabolic panel, were within the normal range, except for a mildly elevated C-reactive protein (CRP) level of 7.8 mg/L (reference range: 0–5.0 mg/L). A non-contrast computed tomography (CT) scan of the head and a CT venogram were normal.

**Figure 1:**
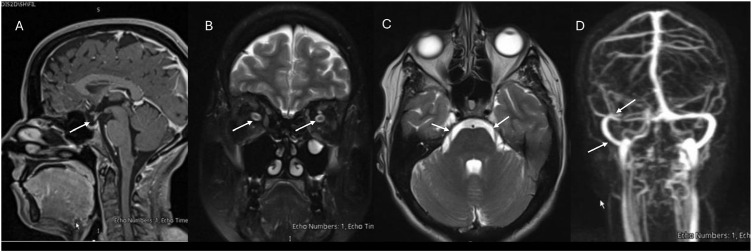
(A) Magnetic resonance imaging (MRI), sagittal view, T1 sequence with intravenous gadolinium showing partial empty sella turcica (arrow). (B) T2 sequence, coronal view showing bilateral optic nerve sheath swelling (arrows). (C) T2 sequence, axial view showing dilated Meckel’s caves (arrows). (D) MRI venogram showing hypoplastic right transverse sinus and mild narrowing in right sigmoid sinus (arrows).

The patient was admitted to the hospital due to a headache with red-flag features, warranting further investigation. Magnetic resonance imaging (MRI) of the head and an MRI venogram showed non-specific signs of increased intracranial pressure without evidence of thrombosis. There was no post-contrast meningeal enhancement (Figure 1).

Follow-up laboratory results at 24 hours showed persistently elevated CRP, and the respiratory viral panel from the nasopharyngeal swab was negative. A lumbar puncture (LP) showed an elevated opening pressure of 30 mmH^2^O. Cerebrospinal fluid (CSF) analysis revealed pleocytosis, with a white blood cell count of 352/uL (reference range: 0–5/uL), 92% lymphocytes, protein concentration of 1.27 mg/L (0.15–0.45 mg/L), glucose level of 2.54 mmol/L (2.2–3.9 mmol/L), an IgG index of 0.7 (0.2–0.6), and negative oligoclonal bands. CSF viral panel was positive for VZV. Mycobacterium tuberculosis PCR (polymerase chain reaction), acid-fast bacilli smear, QuantiFERON test, fungal culture, and cryptococcal antigen were all negative in both CSF and peripheral blood. Serological tests for human immunodeficiency virus (HIV), herpes simplex virus (HSV), and human t-lymphocytes virus (HTLV) were negative. Hepatitis B and C serologies were non-reactive.

A detailed ophthalmologic examination was unremarkable, with no evidence of optic disc swelling. There were no signs of HZ ophthalmicus or retinal necrosis. A diagnosis of VZV meningitis was made, and intravenous (IV) acyclovir therapy was initiated.

Retrospectively, the patient reported that 20 days prior to presentation, she had experienced burning-like pain in the left backside of her head, mainly over the C2–C3 dermatomes, without any rash or vesicles. The pain lasted for four days and resolved spontaneously. Upon further questioning, she also reported having had chickenpox as a child, which supported the diagnosis of ZSH. The patient was treated and discharged safely after receiving intravenous acyclovir (10 mg/kg) every 8 hours for 21 days, followed by a VZV vaccine administered two months later.

## 3. OUTCOME AND FOLLOW-UP

The patient experienced a complete recovery with an uneventful hospital course and resumed her daily activities without any long-term disability or complications during follow-up.

## 4. DISCUSSION

Zoster sine herpete (ZSH) is an uncommon condition in which the varicella-zoster virus reactivates and causes dermatomal neuralgia without an accompanying rash.^1^ This condition is particularly rare in immunocompetent individuals, and its exact prevalence is not well documented as many cases go unrecognized or are misdiagnosed as other forms of neuropathy. However, the incidence of HZ is anticipated to increase in the coming years due to the gradually ageing population and the increasing use of immunosuppressive agents.^3^ Additionally, approximately 95% of young adults are at risk, as indicated by positive VZV IgG serologies.^4^

In this case, the patient was diagnosed with ZSH, complicated by varicella meningitis and increased intracranial pressure. Her case was particularly challenging due to its vague nature, as she did not exhibit typical symptoms of meningitis such as fever, photophobia, or neck stiffness. In retrospect, the distinct dermatomal pattern of burning pain in C2–C3 region drew our attention and led us to suspect ZSH. This highlights the rarity of this case, as most reported cases of ZSH with meningitis present with more classical signs of meningeal irritation.^1,2,5^ This unusual presentation adds to the limited body of literature on ZSH and highlights the need for greater clinical awareness of this rare condition. ZSH is thought to occur either due to the absence of viral migration into the subcutaneous tissue after reactivation^6^ or due to the activation of VZV in the enteric ganglia, whose axons do not extend towards the skin.^7^ In this case, the patient also exhibited increased intracranial pressure, aligning with these pathophysiological mechanisms.

To date, the two gold-standard diagnostic tools are VZV DNA detection with PCR and enzyme-linked immunosorbent assay (ELISA) testing for anti-VZV antibodies, i.e. anti-VZV immunoglobulins (IgG and IgM). PCR is the best first-line test for detecting VZV DNA in the CSF in atypical presentations, as it is rapid, sensitive, and widely available.^7^ The literature emphasizes the importance of PCR as a key diagnostic tool in suspected VZV infections, as noted in a study by S. M. Gregoire et al.^8^ In our case, PCR testing confirmed the diagnosis, aligning with these findings and underscoring its diagnostic value.

Nevertheless, owing to the pathophysiology of VZV reactivation, early in the course of ZSH, the detection of VZV DNA by PCR is more sensitive than the detection of anti-VZV IgG or IgM by ELISA. Conversely, in certain forms of VZV vasculopathy, intrathecal synthesis of VZV-specific IgG is superior to PCR for detecting VZV. In fact, in patients with ZSH whose CSF is negative for VZV by PCR, anti-VZV IgG may still be positive, suggesting a reduced serum-to-CSF ratio of anti-VZV IgG indicative of intrathecal antibody synthesis.^7^

Due to the limited literature and lack of randomized controlled trials on ZSH treatment, no formal guidelines currently exist. It is reasonable to manage ZSH with antiviral agents such as acyclovir or valacyclovir, along with pain management therapy once diagnosed.^9^ In this case, the patient received intravenous acyclovir for 21 days, resulting in a good clinical response and improvement.

It is important to acknowledge the limitations of this case study. Firstly, the findings are based on a single patient, which limits the generalizability to a broader population. Additionally, the retrospective design and reliance on the patient’s medical records and recall may affect the accuracy of the data.

## 5. CONCLUSION

This report contributes to the limited literature on atypical VSV reactivation by demonstrating that ZSH can occur in immunocompetent individuals and presents atypical signs of meningeal irritation and increased intracranial pressure. Physicians should maintain a high index of suspicion for ZSH in such presentations to enable early treatment and improve patient outcomes. The patient in this case was successfully treated without complications. This case highlights the need to establish formal treatment guidelines for ZSH and encourages further research to develop evidence-based management protocols.

## ETHICS APPROVAL AND CONSENT TO PARTICIPATE

This case report was approved by the Medical Research Center at Hamad Medical Corporation (MRC-04-24-477).

## CONSENT FOR PUBLICATION

Written informed consent was obtained from the patient for the publication of this case report, including any accompanying images and photographs. A copy of the written consent is available for review upon request from the Editor of this journal.

## AUTHORS’ CONTRIBUTION

All authors have read, reviewed, and approved the final manuscript.

## COMPETING INTEREST

The authors have no conflicts of interest to disclose.

## AVAILABILITY OF DATA AND MATERIALS

The datasets used and/or analyzed during the current study are available from the corresponding author upon reasonable request.
